# Next-Generation Drugs and Probes for Chromatin Biology: From Targeted Protein Degradation to Phase Separation

**DOI:** 10.3390/molecules23081958

**Published:** 2018-08-06

**Authors:** Katerina Cermakova, H. Courtney Hodges

**Affiliations:** 1 Department of Molecular & Cellular Biology, Center for Precision Environmental Health, and Dan L Duncan Comprehensive Cancer Center, Baylor College of Medicine, Houston, TX 77030, USA; katerina.cermakova@bcm.edu; 2 Center for Cancer Epigenetics, The University of Texas MD Anderson Cancer Center, Houston, TX 77030, USA

**Keywords:** degron, PROTAC, VHL, cereblon, rapamycin, FRB, FKBP, Halo-tag, SNAP-tag, chemically induced proximity

## Abstract

Chromatin regulation is a critical aspect of nuclear function. Recent advances have provided detailed information about dynamic three-dimensional organization of chromatin and its regulatory factors. Mechanisms crucial for normal nuclear function and epigenetic control include compartmentalization of biochemical reactions by liquid-phase separated condensates and signal-dependent regulation of protein stability. Synthetic control of these phenomena by small molecules provides deep insight into essential activities such as histone modification, BAF (SWI/SNF) and PBAF remodeling, Polycomb repression, enhancer looping by cohesin and CTCF, as well as many other processes that contribute to transcription. As a result, a complete understanding of the spatiotemporal mechanisms that underlie chromatin regulation increasingly requires the use of fast-acting drugs and chemical probes. Here, we provide a comprehensive review of next-generation chemical biology tools to interrogate the chromatin regulatory landscape, including selective PROTAC E3 ubiquitin ligase degraders, degrons, fluorescent ligands, dimerizers, inhibitors, and other drugs. These small molecules provide important insights into the mechanisms that govern gene regulation, DNA repair, development, and diseases like cancer.

## 1. Introduction

The organization of chromatin and associated factors is a defining feature of the eukaryotic nucleus. Within living cells, maintenance of chromatin structure is an ongoing process that arises from a dynamic interplay between a highly complex ensemble of nuclear factors. These factors place, remove, and act on chromatin-based moieties, such as histone post-translational modifications and DNA sequence features. Over the last decade, genetic and genomic approaches have identified many essential nuclear factors that mediate key regulatory structures and activities. Moreover, epigenomic studies have provided detailed information about chromatin spatial organization at multiple scales ranging from whole chromosome structures to interactions across a few kilobases [1,2,3,4,5,6]. Together, genome-wide studies have enabled detailed annotation of the major chromatin regulators and interaction sites that contribute to human development and disease.

**Figure 1:**
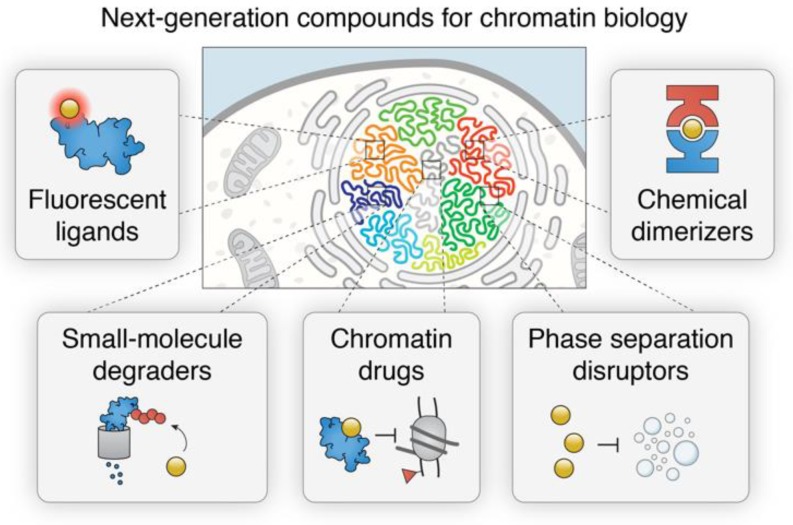
The chemical biology toolkit for investigating chromatin. New developments in chemical biology have yielded powerful new molecules to probe chromatin structure and dynamics.

The fast-moving field of chromatin biology has become a rich source of new drug targets and provided countless opportunities for medicinal chemists and chemical biologists to develop molecular probes specific for chromatin-associated factors (Figure 1). In this regard, several important chromatin regulator targets have recently been identified, including enzymes that covalently modify DNA and histones [7], ATP-dependent chromatin remodelers [8,9], transcriptional repressors [10], transcriptional activators [11,12,13], nuclear hormone receptors [14,15], and others. Because these factors play essential roles in cancer and other disorders [16,17], development of new drugs to dissect their function and target these vulnerabilities represents currently a vibrant area of research.

Several concepts and principles that underlie essential chromatin regulatory activities have long been familiar to chemists, such as phase separation and steady-state equilibrium. Emerging reports are revealing that phase-separated condensates play important roles for spatial organization of several chromatin regulatory activities [18,19,20]. The liquid-phase separation of factors into membrane-less organelles has now been established as a major mechanism to selectively partition bio-macromolecules in the nucleus [21]. Additionally, gain or loss of chromatin readers, writers, and erasers alters the pseudo steady-state equilibrium of chromatin markers. Disruption of this steady-state balance contributes to disease, and can also be exploited for therapeutic benefit. The regulation of these and other features is crucial to maintain efficient cellular function.

A major challenge for the foreseeable future is to identify the spatiotemporal rules that govern these biological activities within the native chromatin environment. Despite a dramatic expansion of our understanding of nuclear regulatory processes acting along the one-dimensional sequence axis, a complete picture of chromatin spatiotemporal regulation in 4D (*x*, *y*, *z*, and time) remains elusive for the following reasons: (1) the long delay between genetic perturbation and experimental measurement does not permit study of kinetics nor allow for detection of immediate downstream effects, (2) genetic alterations are irreversible, which does not permit examination of memory, and (3) the slow temporal resolution of genetic techniques obscures detection of fast activities on order of minutes to seconds and therefore fails to capture the broad class of transient interactions.

Fortunately, new chemical probes and other tools enable characterization of highly dynamic, fast and often heterogeneous processes beyond ensemble- and time-averaged populations. Hence, small molecules are the ideal tools to study fast processes, since they allow specific and rapid perturbation. Here, we review the emerging chemical biology tools developed to study rapid heterogeneous processes that exert transcriptional control of genes and their regulatory elements.

## 2. Small Molecules That Target the Chromatin Landscape

**Figure 2:**
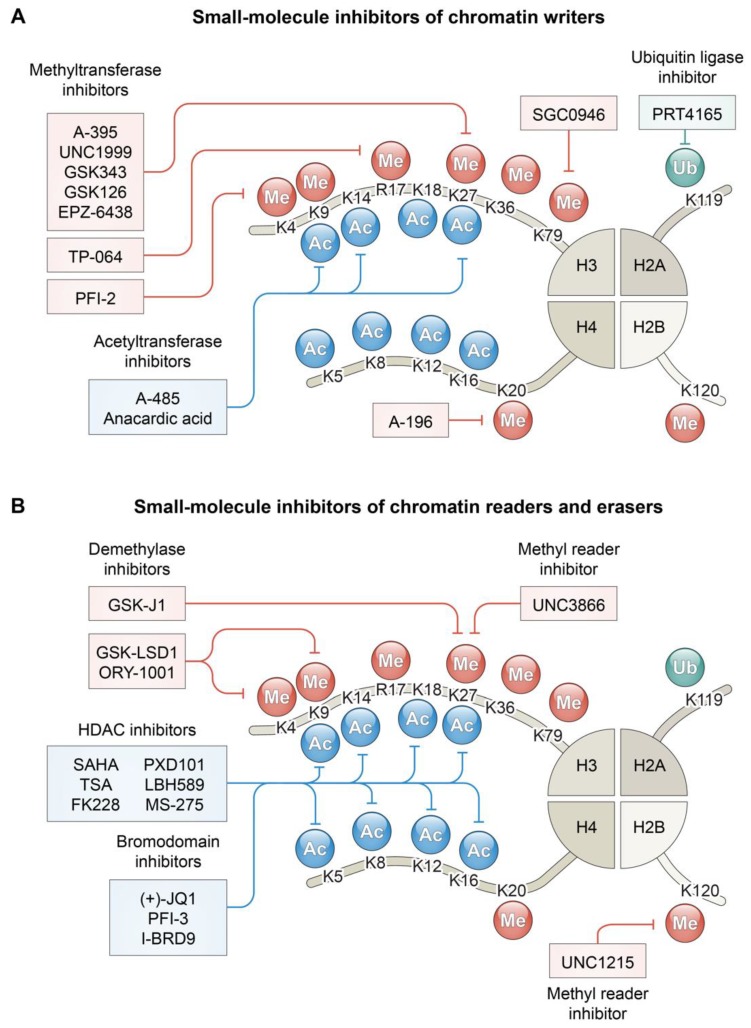
Small-molecule inhibitors of chromatin modifiers. (**A**) Drugs and chemical probes targeting writers of methyl, acetyl, and ubiquityl marks on histone tails; (**B**) Drugs and chemical probes targeting readers and erasers of these epigenetic marks.

High-throughput sequencing methods have transformed our understanding of cell physiology, development, and the origins of diseases like cancer. Among other important advances, these methods have enabled detailed examination of patient tissue samples and cancer cell lines. Genome-wide methods have revealed numerous alterations in the chromatin landscape and uncovered widespread epigenetic deregulation in a variety of malignancies. In many cases, the disruption of chromatin regulatory networks is a major driving event for carcinogenesis [22,23]. Therefore, small molecules that permit controlling the activity of chromatin regulators (Figure 2) have huge potential as anticancer drugs.

In malignant cells, chromatin modifying enzymes and other epigenetic effectors are often disrupted by mutations, deletions, or overexpressed. As a result, the chromatin landscape of these cells undergoes dramatic changes that allow survival and uncontrolled proliferation. Covalent chromatin modifications are very stable. However, unlike irreversible mutations, these changes are reversible. Consequently, there is growing interest in drugs that inhibit activity of chromatin modifiers and specifically reprogram the epigenetic state of malignant cells towards a normal state. One of the first reports that linked altered covalent chromatin modifications to cancer development described DNA hypomethylation in colorectal cancer [24]. DNA hypomethylation increases chromosomal and genomic instability, leading to further oncogenic events and is now recognized as general feature of many malignancies [25]. High-throughput DNA-methylation profiling techniques uncovered that global hypomethylation is accompanied by increased promoter methylation of genes that are established tumor suppressors (such as BRCA1 [26,27], p16/INK4a [28], RASSF1A [29], and MLH1 [30]) and results in their silencing in different cancer types [31,32,33,34]. 

The establishment and maintenance of genomic DNA methylation patterns is catalyzed by the family of DNA methyltransferases (DNMTs). The activity of DNMTs was successfully targeted by nucleoside analogues, which incorporate into newly synthesized DNA and RNA and reduce genomic DNA methylation. The cytidine derivatives 5-azacytidine (Vidaza) and decitabine (Dacogen) are to date among the most successful epigenetic anticancer drugs used in clinics (Table 1). These nucleoside analogues show significant efficacy in hematologic malignancies, specifically in acute myeloid leukemia and myelodysplasia [35,36]. Although new inhibitors of DNMTs have been developed, none has yet to replace 5-azacytidine and decitabine. The most promising next-generation DNMT inhibitors with anti-tumor activity and improved chemical properties are zebularine and guadecitabine [37,38]. While zebularine induced significant toxicity in primates during pre-clinical evaluation and was stopped from entering clinical trials [39], guadecitabine is currently being tested in phase III trials for treatment of several hematologic malignancies, in phase II trials for solid tumors, and many others including several combination therapies [40]. Additionally, several non-nucleoside DNMT inhibitors have been discovered (e.g., nanaomycin A or RG108), however, their current primary application is for research proposes.

Enzymes that regulate chromatin topology also play important roles in human health. This is especially true for highly proliferative cancer cells, where DNA topology is highly regulated during replication. For this reason, drugs that target DNA topoisomerase II (TOP2A and TOP2B) are currently in wide clinical use [90]. TOP2A relaxes supercoiled DNA and is essential for chromatin folding, facultative heterochromatin formation, and gene expression [91,92,93]. The most potent inhibitors of this enzyme are doxorubicin, daunorubicin, ICRF-193, and etoposide, which interfere with DNA synthesis and chromosome segregation through poisoning of type II topoisomerases [44,45,46,47]. These compounds have broad application with demonstrated activity against various malignancies, and are often used in combination with other chemotherapy agents [90].

In addition to DNA modification and topology, histone post-translational modification also plays essential chromatin regulatory roles. Histone tails are decorated with a diverse array of covalently bound marks including lysine and arginine methylation, lysine acetylation, serine and threonine phosphorylation, sumoylation, ubiquitination, and other less conventional modifications. These marks normally govern changes in chromatin structure and compactness, and influence transcriptional repression or activation. Although a number of these histone modifications play important role in deregulation of gene expression, loss of acetylation and methylation of specific histone residues are the two major disruptions that have been associated with pathological changes in cancer cells [94,95]. 

Cells contain a broad palette of enzymes (referred to as chromatin writers and erasers) that keep the epigenetic landscape in balance, as well as effector proteins that are able to recognize the marks through specialized reader modules [7]. A comprehensive set of biologically active small-molecule probes of various chemotypes with well-defined mode of action and selectivity profiles is currently available (see Table 1, Figure 2, and Shortt et al. for review [96]). The development of epigenetic drugs is moreover accelerated by the Structural Genomic Consortium (SGC). Since the toolbox of chemical probes for epigenomics is extensive, we highlight here a selection of important recent advances in this area.

A wide range of structurally diverse histone deacetylase (HDAC) inhibitors that differ in terms of function and specificity is currently available. This breadth serves as a great research tool to dissect the function of these chromatin-associated factors (see Figure 2 and Table 1) [7]. More importantly, many of these agents have demonstrated promising anti-tumor activity, particularly in combination with other anti-cancer drugs, and are currently used in clinics or are in pre-clinical development. While the exact mechanisms behind the antitumor properties of these drugs are in many cases unclear, vorinostat (SAHA) [56], romidepsin (FK228) [53], and belinostat (PXD101) [52,97] have been approved by FDA for T-cell lymphoma and panobinostat (LBH589) [51] for multiple myeloma. Other HDAC inhibitors are in clinical trials for the treatment of hematological and solid malignancies (Table 1) [55,98,99]. Interestingly, HDAC isoforms with cytoplasmic roles and non-chromatin substrates like HDAC8 and HDAC6 also present potential anti-cancer therapeutic targets. In this regard, HDAC8 inhibition by PCI-34051 demonstrated selective toxicity for T-cell over B-cell lineage lymphoma [49] and selective, orally bioavailable HDAC6 inhibitor ACY-1215 synergizes with proteasome inhibition to delay multiple myeloma progression. ACY-1215 has also recently entered clinical trials [50]. Development of more selective HDAC inhibitors with better target identification and improved therapeutic index is a potential path to increase their efficacy. 

While a diverse set of chemical probes targeting histone deacetylases is currently available, the development of histone acetyltransferase inhibitors has lagged. The activity of several histone acetyltransferases such as p300 and PCAF is inhibited by anacardic acid and its derivatives [59]. Unfortunately, these compounds lack potency and selectivity. Recently, virtual screening yielded C646 and A-485, two catalytic inhibitors of the p300 and CBP histone acetyltransferase domains. While C646 was instrumental in demonstrating feasibility of targeting transcriptional activator-driven malignancies by epigenetic drugs, the low selectivity and cytotoxicity of this compound limits its utility outside of cell culture models [100,101,102,103]. The most promising of histone acetyltransferase inhibitors is A-485. This small molecule selectively inhibits proliferation in lineage-specific tumor types, including androgen receptor–positive prostate cancer and several hematological malignancies with promising results in the mouse models [57]. 

The palette of histone methyltransferase inhibitors is also very broad with several emerging therapeutic agents clinically validated in cancer patients (Table 1). An example of successful inhibitors are small molecules specifically recognizing EZH2, the histone methyltransferase of PRC2, which effectively target aberrant methylation levels in lymphomas and are currently in clinical trials [68]. Additionally, the activity of histone methyltransferases is often enhanced by formation of complexes with other factors, which can be therefore also targeted to interfere with the activity of associated methyltransferase. MI-463 and MI-503 are cell-permeable and orally bioavailable inhibitors of MLL1-MENIN interaction, with high potency against MLL-rearranged leukemia [104]. Similarly, MM-401, a small molecule that interferes with MLL1-WDR5 interaction, displays antileukemic activity [105]. Inhibitors of the H3K27 demethylases JMJD3 and UTX also have growth-suppressive properties in acute lymphoblastic leukemia [106] and antitumor activity in xenograft models of diffuse intrinsic pontine glioma [107]. ORY-1001 is a highly potent and selective KDM1A inhibitor that induces blast cell differentiation, and reduces leukemic stem cell capacity in AML [108]. 

Another group of well-established epigenetic anticancer drugs are the inhibitors of acetyl-lysine readers, with most advanced molecules selectively targeting the reading activity of bromodomains and extra-terminal (BET)-protein family in clinical development (Table 1). More recently, high-affinity chemical probes for non-BET acetyl-lysine reader domains have been developed, including PFI-3 [85], which targets the bromodomains of SMARCA4, SMARCA2 and PBRM1, and I-CBP112 [75], which targets CBP and p300. Additionally, several chemical probes that specifically interfere with H3K27me3 reading capacity of PRC1 were reported [109,110,111]. Chemical probes that selectively target methyl reader activity are rather rare. The most potent inhibitor is currently UNC3866, which binds selectively to the chromodomains of CBX4 and CBX7 [88]. These drugs are invaluable for understanding of Polycomb activity and for evaluation of the therapeutic potential of targeting CBX chromodomains. Another example of such a probe is UNC1215, which binds to the malignant brain tumor (MBT) domain of L3MBTL3 [87]. 

Remarkably, a novel potent chromatin remodeling compound PRT4165 that inhibits Polycomb repressive complex 1 (PRC1)-mediated histone ubiquitin ligase activity was recently developed [89]. This drug represents a unique tool to block ubiquitylation signaling at DNA double-strand breaks. In addition, small molecules targeting neurogenin 2 can convert fibroblasts into functional neurons with high efficiency through epigenetic reprogramming [112,113]. This observation underscores the fact that cellular plasticity and adaptability is important not only for cancer, but also for neurodegenerative and other human diseases.

## 3. Chemical Probes for Inducing Targeted Protein Degradation 

**Figure 3:**
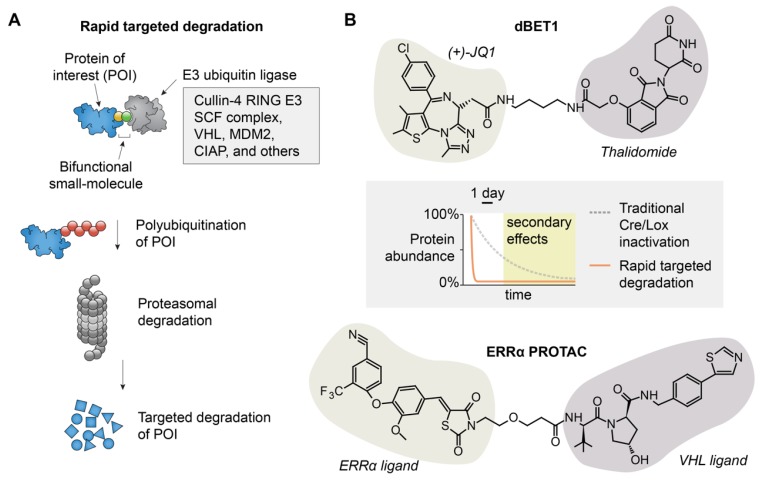
Rapid targeted protein degradation with heterobifunctional small molecules. (**A**) Proteins of interest are linked to E3 ubiquitin ligases. The ensuing polyubiquitination induces rapid proteasomal degradation; (**B**) Example heterobifunctional PROTAC small molecules and comparison of the kinetics of targeted degradation relative to traditional Cre/Lox inactivation.

One of the biggest current challenges in treating human disease is targeting the so-called “undruggable” portion of the proteome. Many validated, highly desirable drug targets—including cancer targets like RAS [114], MYC [115,116], SWI/SNF subunits [81,85], or transcription factors—have historically been considered pharmacologically inaccessible. Targeting these intractable proteins requires innovation and development of new chemical biology tools. An alternative to classic approaches that often focus on modulation of protein interactions or activity is small molecule–induced protein degradation. This strategy combines the advantage of drug-like properties of small molecules with target-specific control of protein abundance (Figure 3). Such systems also have wide utility in basic research for dissection of complex biological systems and downstream pathways since they allow rapid, controllable degradation of target proteins. One particular advantage of this approach is possibility to repurpose drugs that bind their targets with high affinity and selectivity, but did not provide therapeutic effect.

Early attempts to induce protein degradation for therapeutic purposes were based on blocking the molecular chaperone heat-shock protein 90 (HSP90) [117,118]. HSP90 inhibition results in degradation of its client proteins, which are in many cases essential for cell proliferation and survival. Various Hsp90 inhibitors derived from diverse chemical scaffolds have demonstrated potent antitumor activity in a wide range of malignancies, and are currently in clinical or late-stage preclinical investigation [119,120]. However, this approach does not allow degradation of a specific target protein. The first molecules identified to degrade their target protein selectively were estrogen receptor down-regulators (SERDs), targeting specifically estrogen receptor α, a well-known driver of oncogenic signaling in cancer and an established drug target [121]. The most advanced of next-generation SERDs is an orally bioavailable compound GDC-0810 (Brilanestrant), which is currently clinically evaluated in breast cancer patients resistant to standard endocrine therapy [122]. Other orally bioavailable SERDs with high potency have also been described [123,124,125,126,127,128]. Interestingly, similar effects were observed for the selective androgen receptor (AR) down-regulators (SARDs) [129,130,131].

An important class of small molecules that degrade their respective targets without requiring any genetic manipulation is phthalimide-derived drugs also known as immunomodulatory imide drugs (IMiDs). Among the most notable of this class is thalidomide, which was originally used to treat morning sickness, but was banned in the 1960s for causing serious congenital birth defects. Remarkably, thalidomide and its close analogues were repurposed and are currently used as potent anticancer agents [132]. Specifically, CC-5013 (Lenalidomide) is approved for the treatment of relapsed multiple myeloma, myelodysplastic syndrome, and mantle cell lymphoma. It is also in Phase III trials for the treatment of acute myeloid leukemia and chronic lymphoblastic leukemia [133,134]. CC-4047 (Pomalidomide) has been approved for relapsed multiple myeloma [135] and a more recently described compound, CC-122, displays activity as a pleiotropic pathway modifier. CC-122 is in Phase I trials for multiple myeloma, diffuse large B cell lymphoma, chronic lymphoblastic leukemia, and several solid tumors [136].

The efficacy of phthalamide derivatives as anticancer agents has prompted investigation into their mechanism of action. Thalidomide binds to cereblon (CRBN), a substrate receptor of the cullin-4 RING E3 ligase complex, which results in polyubiquitination and degradation of transcription factors Ikaros (IKZF1) and Aiolos (IKZF3) [137,138,139]. Recently, rational design of bifunctional phthalimide-conjugated ligands conferred CRBN-dependent target protein degradation. Specifically, phthalimide conjugation to JQ1 (referred to as dBET1) and FKBP12 ligand (referred to as dFKBP) was leveraged for posttranslational degradation of their respective specific targets, BRD4 and FKBP12 [140]. Since CRBN is ubiquitously expressed, this strategy has broad utility in developmental and disease biology. Induced degradation of BRD4 by dBET1 in vivo resulted in improved antitumor efficacy in a leukemia xenograft model compared with the effects of JQ1. A more general advantage of this approach is in the feasibility of degrading intractable targets using phthalimide-conjugation of target-binding ligands, regardless of whether those ligands possesses target-specific inhibitory activity.

An analogous strategy is applied in systems known as PROTACs. PROTACs are heterobifunctional molecules that have discrete binding moieties for the substrate of interest and for an E3 ligase connected by a chemical linker. Therefore, PROTACs are able to specifically link the target protein and the E3 ligase. As a result, ubiquitin can be transferred from an E2 to the target protein, which is eventually degraded by the proteasome. The main advantage of PROTACs is their versatility. Since various ligands targeting proteins of interest can be used to recruit these proteins to the E3 ligase, and the human genome encodes more than 600 E3 ligases [141], it is possible to develop a vast array of PROTACs for drug discovery. 

The first PROTAC consisted of IκBα phosphopeptide that is recognized by β-TRCP (subunit of Skp1-Cullin-F box protein (SCF) ubiquitin ligase complex), whereas the other domain was composed of methionine aminopeptidase-2 (MetAP-2) inhibitor ovalicin [142]. While the first generation of PROTACs successfully and specifically degraded their targets, they were active in the low-micromolar range with only partial degradation of the protein of interest and had poor cell permeability [142]. Significant advancement of PROTACs was achieved by identification of more specific drug-like binders of different E3 ligases. The poor cell permeability of the first-generation PROTACs was significantly improved by adopting HIF-1α recognition motif to hijack the activity of von Hippel–Lindau (VHL) E3 ligase in the design of the second-generation PROTACs [143,144,145,146,147]. Several series of non-peptide-based binders to the VHL ligase were identified, optimized, and incorporated into PROTACs with more drug-like properties, resulting for the first time in highly potent cellular effects [148]. In addition to β-TRCP and VHL, MDM2 [149] and CIAP [150] have been employed for induced protein ubiquitination using a heterobifunctional dimer approach. 

So far, several oncoproteins, such as androgen receptor, estrogen receptor, ERRα, and BRD4, have been specifically ubiquitinated and destroyed via PROTACs [151]. In addition, PROTACs targeting methionine aminopeptidase-2 (MetAP-2), the aryl hydrocarbon receptor [152,153], and cellular retinoic acid-binding proteins (CRABPs) [154] have also been developed. PROTAC-induced protein degradation has yielded impressive preliminary efficacy in a limited number of cellular and in vivo systems, but its broader utility and application in a clinical setting remains to be evaluated.

**Figure 4:**
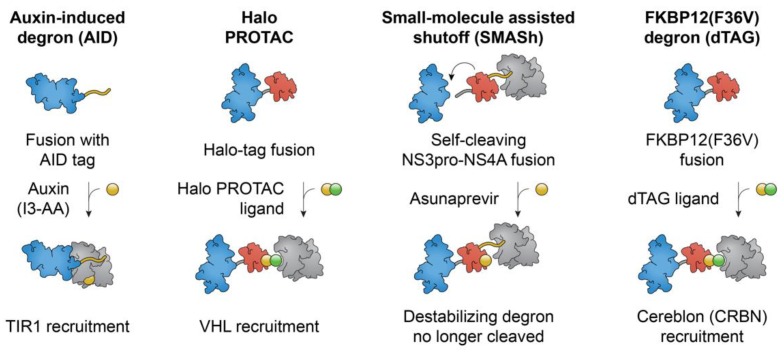
Genetically encoded fusion strategies for targeted protein degradation. A variety of fusion tags exist that are suitable for orthogonal targeted protein degradation strategies. These strategies require recombinant expression in experimental systems.

Several genetically encoded systems also allow for rapid protein degradation in research settings (Figure 4). One such targeted protein degradation system is the auxin-inducible degron (AID). Unlike the above mentioned degrons, AID requires genetic manipulation, which limits its utility in medicine, however, is invaluable for addressing biological questions. The AID system has enabled control of the abundance of a diverse set of targets, including factors which lack selective inhibitors, in transformed and non-transformed mammalian cells [155]. The minimal AID domain fused to the protein of interest is small (44 amino acids) and enables rapid (*t*_1/2_ = 20 min) depletion of the protein of interest [156,157], somewhat faster than PROTAC-based approaches. Moreover, AID-mediated instability is reversible, making this system especially versatile. The AID tag was recently successfully delivered by CRISPR/Cas9 gene editing technology underscoring the possibility to perform acute and reversible conditional depletion of any endogenous protein of interest [158]. This approach was applied for degradation of CTCF to interrogate mechanisms that underlay chromosomal folding and architecture [159].

More recently, HaloPROTACs [160], Small Molecule-Assisted Shutoff (SMASh) degraders [161], and dTAG [162] systems were developed. These approaches allow abundance control of genetically modified fusion-proteins in living cells through orthogonal mechanisms. The Halo tag is a modified *Rhodococcus* dehalogenase able to undergo a self-labeling reaction with cell-permeable alkylchlorides, which is widely used as a fusion tag to bio-orthogonally label proteins in living cells [163,164]. HaloPROTACs leverage VHL E3 ligase ligands conjugated with hexyl chloride to degrade fusion proteins harboring a Halo-tag [160]. Since Halo-tag fusion proteins are readily available reagents commonly used in biological studies, there is a huge potential for application of HaloPROTAC in genetics studies. In the SMASh system, a destabilizing degron is fused to a catalytic fragment of NS3 protease from hepatitis C virus, followed by NS3 cleavage site (NS3pro-NS4A) and gene product of interest [161]. Such fusion proteins are cleaved by the NS3 protease after translation, yielding an unmodified protein of interest and degron-tagged NS3 destined for degradation. However, in the presence of specific NS3 protease inhibitors like asunaprevir, the fusion proteins retain intact and all components are degraded. This system requires minimal modification of the protein of interest and utilizes small molecules with proven safety and bioavailability in mammals. The dTAG platform couples a degrader composed of selective FKBP12^F36V^ ligand AP1867 and thalidomide with expression of FKBP12^F36V^ in-frame with a protein of interest [162]. Importantly, the efficacy of dTAG was demonstrated in a mouse model, supporting the broad utility of this system in biological research.

## 4. Proximity-Inducing Drugs 

**Figure 5:**
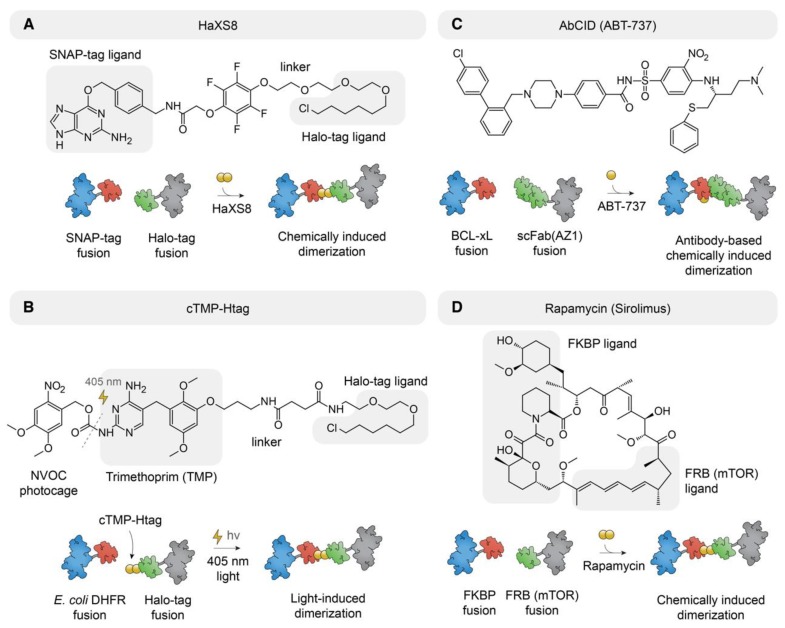
Heterobifunctional small molecules for chemically induced proximity. (**A**) Chemically induced dimerization of SNAP-tag and Halo-tag with HaXS8; (**B**) Antibody-based chemically induced dimerization with AbCID (ABT-737); (**C**) Light-induced dimerization of *E. coli* dihydrofolate reductase (DHFR) and Halo-tag with cTMP-Htag; (**D**) Chemically induced dimerization of FRB and FKBP with rapamycin.

Living cells are complex, plastic and stochastic systems that constantly sense their inner and outer microenvironment and integrate received signals to generate proper biological responses. On the molecular scale, one of the critical aspects that allows cells to produce discrete and specific responses is the physical proximity or distance of two or more molecules. The physical presence and absence of specific regulators provides the cell with a potential to self-regulate its function by adjusting gene expression. Synthetic approaches to induce proximity of given factors are instrumental in investigation of how specific cellular signals generate appropriate physiological responses and how these responses are altered in disease settings. Engineering of chemical probes that induce physical proximity of two bio-macromolecules (see Stanton and Chory et al. for review) [165] enables elucidation of their contribution into regulatory circuits, assessment of their immediate downstream effects, control over their cellular localization or analysis of their kinetic parameters in living cells (Figure 5).

Chemically induced proximity (CIP) is especially valuable for investigation of processes driven by short-lived and transient interactions like signal transduction, transcription, or chromatin remodeling. Perturbations with traditional biochemical approaches have limited temporal resolution, which obscures detection of fast activities, while CIP with drug-like compounds enables rapid perturbations that can be performed in controlled manner. The first synthetic cell-permeable inducer of proximity is a derivate of tacrolimus known as FK1012. This small-molecule induces dimerization of FK506-binding protein (FKBP), a protein folding chaperone with peptidyl-prolyl cis/trans isomerase activity. FKBP dimerization results in signal transmission and specific target gene activation in T-lymphocyte transduction pathway [166]. Similar approaches leveraging FK1012-triggered dimerization of FKBP fused to different nuclear factors were later used to investigate other pathways, for example, Fas signaling in apoptosis or Ras/Raf interplay in MAPK cascade [167,168]. Chemical dimerizers of other domains were also described including HaXS dimerizer [169], a heterodimerization system covalently linking Halo-tag and SNAP-tag with high selectivity and intracellular reactivity, cTMP-Htag [170], a photocaged dimerizer enabling reversible light-induced recruitment of eDHRF (*E. coli* DHFR) tagged protein to a Halo-tagged protein, or AbCID [171], a dimerizer approach that triggers antibody recognition of a chemical epitope formed only upon binding of a small molecule by the target factor. In addition, the plant phytohormone S-(+)-abscisic acid pathway has also been engineered to control the proximity of cellular proteins [172]. This system was applied to investigate the temporal order of chromatin-based processes like histone acetylation or gene activation [173].

Arguably, the most notable chemical hetero-bifunctional dimerizer is rapamycin (sirolimus). Rapamycin directly binds to FKBP12 and FRB domain from mTOR Complex 1 and chemically induces their association. The prime application of this system in research is the initiation of rapid rapamycin-dependent interaction of two factors fused to FKBP and FRB, and subsequent monitoring of ensuing effects. Induced proximity has been used to investigate heterochromatin formation by recruitment and spreading of HP1 [174,175,176], as well as rapid eviction of Polycomb repressive complexes at bivalent genes by BAF ATP-dependent remodeling complexes [177]. Similar approaches were applied to induce DNA demethylation by TET2 [178]. This system was also instrumental in studying secretory mechanisms of Golgi and endoplasmic reticulum during the cell cycle [179], and in examination of synaptic transmission [180]. 

Rapamycin has widespread utility in medicine as an immunosuppressant to prevent organ transplant rejection, since it inhibits the activation of immune cells by reducing their sensitivity to interleukin-2 through mTOR inhibition. Moreover, the antiproliferative effect of rapamycin is currently being leveraged for breast cancer therapy [181]. In addition, this approach holds great promise for providing novel therapeutic applications in CAR T-cell therapy, where chemically induced proximity-based safety switches were recently incorporated to trigger apoptosis of CAR T cells [182]. 

## 5. Small Molecules for Investigation of Liquid Phase Separation 

Liquid-phase separation is emerging as a common biophysical basis underlying many important cellular processes [183,184,185]. Phase-separated assemblies comprised of heterogeneous liquid-like mixtures of proteins and nucleic acids provide a fundamental regulatory mechanism to compartmentalize the intracellular space. Consistently, several membrane-less organelles exhibit a concentration threshold for assembly and ability to undergo fission and fusion, which are hallmarks of phase separation. Structures that behave as membrane-less organelles include nuclear bodies such as nucleoli involved in ribosome biogenesis, transcription factories associated with active RNA polymerase II, as well as Polycomb group bodies, HP1 pericentric heterochromatin foci, and others [18,186,187,188,189]. The barrier-free character of these condensates allows for rapid exchange of components with the surrounding environment, and rapid alteration of their internal equilibrium [20,190,191]. The tight regulation at discrete foci located throughout the nucleus is crucial to maintain efficient cellular functions including heterochromatin compaction [187,192], stress granule formation [193], splicing [194,195], super-enhancer activity [20,196], and many others. Biological condensates are frequently observed during cell division and development, where cellular processes are under stringent regulation [185]. At the molecular level, phase separation is largely driven by the presence of many weak, transient interactions between molecules with multivalent domains or intrinsically disordered regions [197,198]. In cells, condensation of liquid phase–separated assemblies can be regulated by active processes, including transcription and various posttranslational modifications [199,200,201].

Understanding how sequence-encoded information of proteins and nucleic acids drives coexistence and the physicochemical properties of these diverse condensates is essential for deciphering the regulatory logic embedded in the genome. Moreover, the formation and physical properties of membrane-less compartments are of great importance, because transitions into more solid-like states have been linked to age-related diseases [202,203]. Unfortunately, the characterization of these assemblies remains difficult, because liquid-like condensates and solid-like aggregates are morphologically very similar and cannot easily be discriminated by fluorescence microscopy. Current approaches to determine the properties of phase-separated assemblies in living cells are assessing the sphericity, analyzing fusion events or measuring fluorescent recovery after photo bleaching (FRAP) [204]. Because the compartments under investigation are often very small and their mobility in cells is quite high, fusion and FRAP recovery are often very fast.

**Figure 6:**
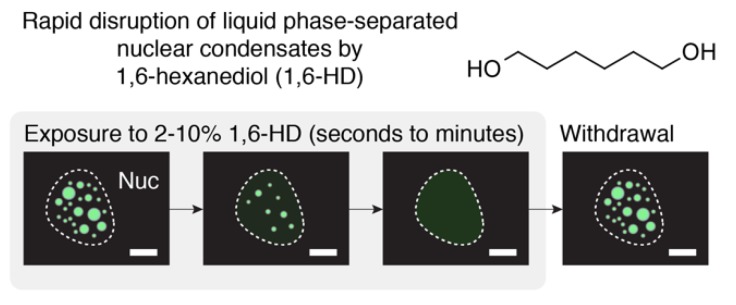
Disruption of phase-separated nuclear condensates with 1,6-hexanediol (1,6-HD). A hallmark of phase-separated nuclear condensates is sensitivity to 1,6-HD. As illustrated in this cartoon model, condensates typically shrink and disappear within seconds to minutes upon incubation with 2–10% 1,6-HD. Withdrawal of 1,6-HD rapidly restores dynamic condensate structure in the nucleus (Nuc).

The aliphatic alcohol 1,6-hexanediol is the only small molecule that is currently used to distinguish liquid-like and solid-like assemblies in vitro as well as in living cells (Figure 6). Hexanediol dissolves dynamic, liquid-like assemblies, such as P bodies, transcription factories, or RNA-protein granules in living cells [196,200,205]. In contrast, solid-like bodies, such as protein aggregates and cytoskeletal assemblies are largely resistant to hexanediol. It is not yet understood how hexanediol affects liquid-like assemblies. One possible explanation is that hexanediol inhibits the formation of a liquid protein phase by interfering with the weak interactions between proteins, DNA, or RNA. Unfortunately, extended exposure of yeast and mammalian cells to hexanediol is cytotoxic and causes abnormal changes in cell morphology, which trigger the formation of aberrant assemblies [200,206]. 

Many well established small-molecule inhibitors are currently utilized to characterize involvement of distinct factors in liquid droplet formation and to investigate whether a phase-separation model is consistent with nuclear function. JQ1, a specific inhibitor of BRD4, was used to demonstrate that efficacy of super-enhancer-mediated gene regulation can be explained by their presence in phase-separated multi-molecular assemblies. 5,6-Dichlorobenzimidazole ribofuranoside (DRB), a reversible RNAP2 CTD kinase inhibitor, was leveraged to investigate assembly and maintenance of paraspeckles [207]. Moreover, the glycolysis inhibitor 2-deoxyglucose and respiratory chain inhibitor antimycin A have been used to dissect Pab1-marked stress granule formation [208]. Although these drugs are instrumental for our understanding of liquid-phase separation in nuclear function, new chemical probes for more selective investigation of these assemblies would be of significant interest to the biology community.

## 6. Fluorescent Ligands for Direct Visualization of Chromatin Factors in Living Cells

The nucleus is a highly dynamic environment that constantly and rapidly adapts in response to diverse signals. A large fraction of the nuclear processes is governed by short-lived interactions that are often poorly assayed by traditional approaches. The detection of transient interactions currently remains a major frontier in chromatin biology. Live-cell imaging offers unprecedented opportunities for direct observation of chromatin-associated factors inside living cells and for quantitative assessment of the principles that govern their function. Current super-resolution microscopy techniques overcome the loss of information due to population averaging. These methods enable investigation of kinetic properties (e.g., on- and off-rate constants, residence time distributions, and bound fraction), heterogeneity, spatiotemporal distribution and stochasticity of diverse molecular events at the single-molecule level (see Liu et al. for review) [209]. Recently, direct measurements of the mobility of chromatin-related factors in living cells have provided valuable insights about the mechanisms that underlie fundamental processes in development and malignancy [210,211,212].

The spatiotemporal resolution sufficient for tracking movement of individual molecules in the nucleus was achieved through implementation of new microscopy methods that limit the illumination volume, like highly inclined and laminated optical sheet (HILO) microscopy [213] or Bessel beam selective plane illumination [214,215]. However, perhaps the most critical aspect of these techniques is engagement of highly photostable and bright fluorescent chemical probes. Although single-molecule imaging experiments can be performed with fluorescent proteins, they have less favorable photophysics compared to available organic dyes. The utility of organic fluorescent dyes was advanced by their coupling to self-labeling protein tags (e.g., Halo-tag [163,164], or SNAP-tag [216]). Available commercial organic fluorescent probes such as Cy3, Cy5, Alexa Fluor 555, Alexa Fluor 647 ATTO 655 or ATTO 647 span the visible spectrum and can be specifically conjugated to almost any molecule of interest through diverse labeling strategies [209]. These fluorophores possess appropriate brightness and photostability for single-molecule experiments; however, many of these dyes exhibit poor cell permeability and therefore are more appropriate for labeling in vitro or in fixed cells.

**Figure 7:**
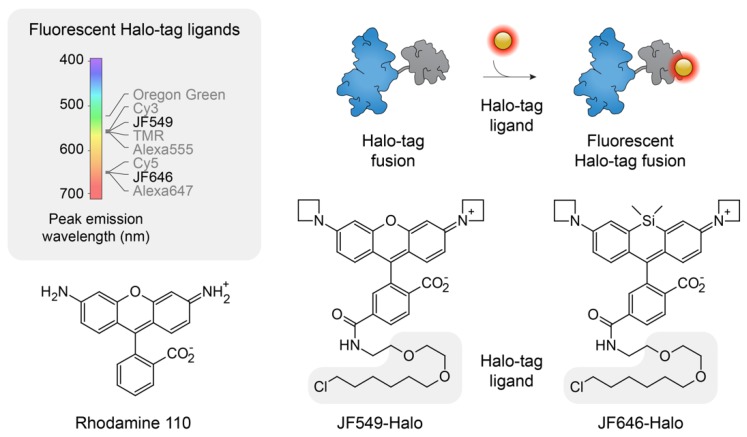
Fluorescent ligands for direct visualization of protein dynamics in native chromatin. Improved photostability of rhodamine-based dyes and conjugation with chloroalkane reagents has resulted in bright, photostable, cell-permeable fluorescent dyes compatible with the Halo-tag system.

For tracking experiments inside living cells, derivatives of natural fluorophores from rhodamine family such as tetramethylrhodamine (TMR) or rhodamine 110 have been widely used. These dyes exhibit high solubility and cell permeability, however their quantum efficiency and brightness is suboptimal. Replacement of the dimethylamino groups in TMR with four-membered azetidine rings doubled the quantum efficiency and improved the photon yield, while preserving the spectral properties and excellent cell permeability. Together these improvements resulted in the dye known as Janelia Fluor 549 (JF549) [217]. This strategy was extended to red-shifted rhodamine analogs, such as the silicon-containing JF646, allowing multicolor experiments and imaging with longer, less damaging wavelengths [217]. These dyes were successfully combined with Halo-tag ligand to efficiently cross the membrane and selectively label Halo-tagged fusion proteins [217] (Figure 7). In addition, photoactivatable versions of JF549 and JF646 have been reported, allowing sophisticated PALM and STORM single-particle tracking experiments [218]. Despite enormous progress in development of fluorophores for live-cell imaging, several features would push forward current limits of super-resolution imaging, notably additional dyes in blue and green spectral ranges, improved photostability, and increased brightness. Coupling these new tools with complementary microscopy approaches has great potential to advance our understanding of 4D spatiotemporal dynamics within the nucleus [219,220].

## 7. Conclusions

Emerging chemical biology probes controlling activity, stability, or localization of nuclear factors are invaluable tools to further our mechanistic understanding of dynamic chromatin organization. These tools also permit entirely new investigations into the divergent roles of chromatin-associated factors in healthy and diseased cells. Small molecules that permit controlling the activities of deregulated epigenetic modulators and effectors have great potential as anticancer drugs, with several examples currently in clinical use or in trials. In particular, conditional elimination of pharmacologically inaccessible disease-promoting proteins has enormous potential; these molecules are increasingly explored for clinical application and serve as invaluable research tools. The design of drugs that can selectively target specific liquid phase-separated condensates is also of high interest for the broad scientific community as well as for the pharmaceutical industry, since specific delivery into these assemblies might improve efficacy and minimize toxicity of new compounds. Finally, chemically induced proximity is especially valuable for investigation of processes driven by short-lived, transient interactions, many of which contribute greatly to human health. Altogether, continued development of selective compounds that control the epigenomic landscape holds great promise as research tools and for targeted, more precise therapies.

## Figures and Tables

**Table 1:** Potent and selective drugs to inhibit chromatin regulators.

Molecule	Protein Target (s)	Associated Chromatin Feature	IC50/EC50	References
DNA methyltransferase (DNMT) inhibitors				
guadecitabine (SGI-110)	DNMTs	DNA methylation	–	[41]
Zebularine	DNMTs	DNA methylation	–	[39,42,43]
5-azacytidine (Vidaza)	DNMT1, DNMT2, and RRM2	DNA methylation	–	[35]
decitabine (Dacogen)	DNMT1	DNA methylation	–	[36]
Topoisomerase inhibitors				
doxorubicin	Type II topoisomerases	Topology	–	[44]
daunorubicin	Type II topoisomerases	Topology	–	[45]
ICRF-193	Type II topoisomerases	Topology	–	[46]
etoposide	Type II topoisomerases	Topology	–	[47]
Histone deacetylase (HDAC) inhibitors				
MS-275	HDAC1-3	histone acetylation	510 nM	[48]
PCI-34051	HDAC8	Cytoplasmic	10 nM	[49]
ACY-1215	HDAC6	Cytoplasmic	5 nM	[50]
LBH589 (Panobinostat)	HDAC classes I, II, and IV	histone acetylation	5 nM	[51]
PXD101 (Belinostat)	HDAC classes I, II, and IV	histone acetylation	27 nM	[52]
FK228 (Romidepsin)	HDAC class I	histone acetylation	47 nM	[53,54]
Trichostatin A	HDAC classes I, II	histone acetylation	20 nM	[55]
SAHA (Vorinostat, Zolinza)	HDAC classes I, II, and IV	histone acetylation	10 nM	[56]
Histone acetyltransferase inhibitors				
A-485	CBP (p300)	histone acetylation	3–10 nM	[57,58]
anacardic acid	p300, PCAF	histone acetylation	24 nM	[59]
Histone demethylase inhibitors				
GSK-LSD1(ORY-1001)	LSD1 (KDM1A)	H3K4me, H3K9me	<5 nM	[60]
GSK-J1	JMJD3, UTX, JARID1B	H3K27me	9 µM	[61]
Histone methyltransferase inhibitors				
MRK-740	PRDM9	H3K4me3	800 nM	SGC ^1^
SKI-73, SKI-72	PRMT4 (CARM1)	H3R17me	1.3 µM	SGC ^1^
SGC3027	PRMT7	H4R3me2	<2.5 nM	SGC ^1^
BAY-6035	SMYD3	H3K4me2,3, H4K5me	70 nM	SGC ^1^
PFI-5	SMYD2	H3K4me1,-2,-3	900 nM	SGC ^1^
LLY-283	PRMT5	H4R3me, H3R8me	25 nM	SGC ^1^
TP-064	PRMT4	H3R17me	43 nM	[62]
A-395	EED (PRC2 complex)	H3K27me2	90 nM	[63]
A-196	SUV420H1, SUV420H2	H4K20me	500 nM	[64]
GSK591	PRMT5	H4R3me, H3R8me	56 nM	[65]
MS049	PRMT4, PRMT6	H3R17me, H3R2me	970 nM	[66]
OICR-9429	WDR5 (interacts with KMT2A)	H3K4me	233 nM	[67]
UNC1999	EZH2 (PRC2 complex)	H3K27me3	124 nM	[68]
PFI-2	SETD7	H3K4me	100 nM	[69]
SGC0946	DOT1L	H3K79me	10 nM	[70]
GSK343	EZH2	H3K27me3	174 nM	[71]
GSK126	EZH2	H3K27me3	9.9 nM	[72]
EPZ-6438 (Tazemetostat)	EZH2	H3K27me3	11 nM	[73]
MI-463	MENIN (interacts with KMT2A)	H3K4me	15 nM	[74]
MI-503	MENIN	H3K4me	14 nM	[74]
Histone acetyl reader inhibitors				
I-CBP112	CBP (p300)	H3K18ac	5 µM	[75]
L-Moses	PCAF (GCN5)	H3K9ac	126–600 nM	[76]
GSK4027	PCAF (GCN5)	presumed acetyl-lysine	60 nM	[77]
BAY-850	ATAD2	presumed acetyl-lysine	1 µM	[78]
GSK8814	ATAD2, ATAD2B	presumed acetyl-lysine	2.7 µM	[79]
GSK6853	BRPF1B	presumed acetyl-lysine	20 nM	[80]
TP-472	BRD9, BRD7	presumed acetyl-lysine	320 nM	[81,82]
BAY-229	BRD1, TAF1	presumed acetyl-lysine	<13 nM	[83]
I-BRD9	BRD9	presumed acetyl-lysine	159 nM	[84]
PFI-3	SMARCA4, SMARCA2, PBRM1 (BAF and PBAFcomplexes)	presumed acetyl-lysine	1 µM	[85]
(+)-JQ1	BRD4, BET family	presumed acetyl-lysine	33–77 nM	[86]
Histone methyl reader inhibitors				
UNC1215	L3MBTL3	H3K20me	40 nM	[87]
UNC3866	CBX4/CBX7 (PRC1 complex)	H3K27me3	66 nM	[88]
Histone ubiquitin ligase inhibitor				
PRT4165	RING1A, RING1B (RNF2), (PRC1 complex)	H2AK119ub	3.9 µM	[89]

^1^ Reference unavailable at time of writing but molecule available through the Structural Genomics Consortium (SGC).
